# Sensorimotor circuitry involved in the higher brain control of coughing

**DOI:** 10.1186/1745-9974-9-7

**Published:** 2013-03-06

**Authors:** Stuart B Mazzone, Alice E McGovern, Seung-Kwon Yang, Ariel Woo, Simon Phipps, Ayaka Ando, Jennifer Leech, Michael J Farrell

**Affiliations:** 1 School of Biomedical Sciences, University of Queensland, St Lucia, Brisbane, QLD, 4072, Australia; 2 The Florey Institute of Neuroscience and Mental Health, Melbourne, VIC, 3010, Australia

## Abstract

There is an overwhelming body of evidence to support the existence of higher brain circuitries involved in the sensory detection of airways irritation and the motor control of coughing. The concept that cough is purely a reflex response to airways irritation is now superseded by the recognition that perception of an urge-to-cough and altered behavioral modification of coughing are key elements of cough disorders associated with airways disease. Understanding the pathways by which airway sensory nerves ascend into the brain and the patterns of neural activation associated with airways irritation will undoubtedly provide new insights into disordered coughing. This brief review aims to explore our current understanding of higher order cough networks by summarizing data from recent neuroanatomical and functional studies in animals and humans. We provide evidence for the existence of distinct higher order network components involved in the discrimination of signals arising from the airways and the motor control of coughing. The identification of these network components provides a blueprint for future research and the development of targeted managements for cough and the urge-to-cough.

## John Widdicombe remembered

In 2002, while holidaying in London, I (SBM) received a pleasant email invitation to join John and his wife Margaret for dinner in a little Italian restaurant owned by a family member. Over dinner John and I chatted about many things ‘respiratory’, including the complete absence (at the time) of any neurophysiological insights into the control of cough by supramedullary brain regions. John described his disappointment at several failed experiments with Abe Guz, trying to image brain responses during the urge-to-cough in humans. By the end of that night John had set me a friendly challenge, to move beyond the brainstem and tease apart the complexities of cough control in the higher brain. This short preface is to acknowledge John’s support and encouragement for the work that is described below and to pay tribute to his immense contribution to the field. It has been an honor and a privilege for our team to contribute to this John Widdicombe memorial series, in memory of a truly inspirational respiratory physiologist.

## Evidence for higher brain involvement in coughing

Over the past decade there has been a significant increase in research into cough neural pathways and this has provided respiratory researchers with new insights into the cough reflex as well as the cognitive and behavioral aspects of respiratory defensive responses mediated by sensory neural activation [reviewed in [1-5]. We have long suspected that higher brain neural pathways are involved in the perception of airway irritation and the behavioral modification of coughing, yet attempts to study this have only recently begun to appear in the scientific literature. However, we are now beginning to appreciate the very important role that higher brain circuits play in both the ongoing control of respiration as well as in manifestations of respiratory pathophysiology, particularly in the generation of symptoms associated with pulmonary diseases. This brief review will summarize studies from our group and others which are beginning to describe the identity and organization of the higher brain sensorimotor circuits that regulate coughing.

Cough is a motor act, and is characterized by reorganization of the central breathing pattern generator to produce the characteristic three phases of a typical cough (inspiration, compression and expiration). It can be evoked reflexively or enlisted voluntarily, indicating that multiple inputs can drive the final motor response [6,7]. Reflex cough is largely dependent upon vagal afferent inputs that are processed at the brainstem level [6,8-10]. Such reflex evoked coughing can be elicited from the airways under general anesthesia or in decerebrate animals suggesting that neural processing above the level of the brainstem is not essential for reflex coughing [6,8]. Thus, it seems likely that reflex cough represents the basic defensive mechanism for clearing the airways of an acute respiratory insult, ensuring that airway patency is maintained. Much of the primary afferent and brainstem processing network involved in reflex cough has been described in detail elsewhere (see references above) and is therefore not the subject of the present review. Voluntary cough and cough suppression, on the other hand, involve higher brain circuitries that are responsible for planning and initiating the motor act and for consciously controlling the final motor output to the muscles of respiration [7,11]. Furthermore, although reflex cough does not require suprapontine involvement, higher level regulatory mechanisms exist that *can* provide modulatory inputs to the basic brainstem reflex circuit [7].

In addition to higher brain descending pathways that regulate the motor act of coughing, it is now well established that higher order sensory pathways receive inputs from the airways and play an important role in generating cognitive sensory responses to airways irritation [12-14]. The urge-to-cough is a sensory experience that provides an awareness of the presence of an irritation in the upper airways and in turn drives the resultant desire to respond to that stimulus by coughing (to facilitate clearance of the offending irritant). Accordingly, the urge-to-cough can be considered one of several pulmonary sensory mechanisms that allows for the conscious perception of the operating conditions of the respiratory system and for the behavioral regulation of respiration in order to respond to changes in these conditions [2]. In this sense, cough driven by the urge-to-cough is clearly distinct from reflex coughing, in which the conscious behavioral component is minimal or non-existent. Given that it is becoming more widely accepted that chronic cough in disease has a significant behavioral component, it stands to reason that future therapeutic strategies for relieving excessive coughing will be underpinned by an understanding of the neural basis of the urge-to-cough.

## Suprapontine cough pathways

**Figure 1:**
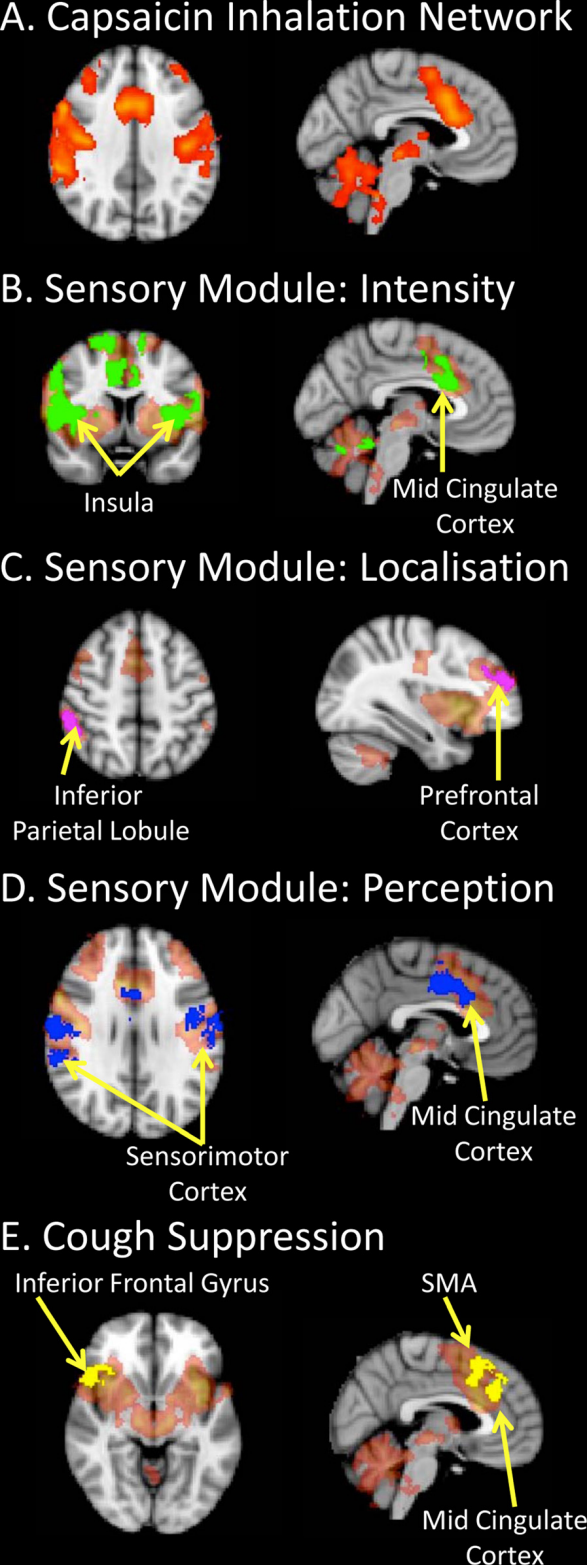
**Functional brain maps of sensorimotor activations following capsaicin inhalation in humans.** (**A**) Capsaicin inhalation is associated with the activation of a distributed network in the brain [13]. We propose that this network is composed of several sub-circuits (modules) involved in sensory discrimination and motor control (panels **B**-**E**). Our published data indicate that discrete regional responses incorporate modules that (**B**) encode stimulus intensity, (**C**) identify stimulus location, (**D**) determine perceptual experiences and (**E**) can suppress evoked motor responses (see [7,12]). The module specific activations shown in green, pink, blue and yellow on panels **B**-**E** are superimposed on the distributed capsaicin inhalation network (shaded orange) as highlighted in panel **A**. See cited references for full details of experimental design, data analysis and interpretation.

Humans (and probably other mammals) can perceive, and to some extent localize, an irritation that is present in their airways. It is this perceptual awareness that results in the unpleasant sensations arising from the airways during irritation or inflammation (for example, the persistent laryngeal itch experienced during an upper respiratory viral infection), and ultimately drives the urge-to-cough [15]. Furthermore, humans upon voluntarily command can evoke or suppress a cough, or can have their coughs *subconsciously* modified by higher order brain processes involving placebo, anxiety or attentional tasks [7,16-18]. Taken together these observations support the existence of neural circuitry above the level of the reflex processing sites in the brainstem that receive inputs from the airways and provide descending control over, or in parallel to, the medullary cough pattern generator [7]. We have used a combination of functional brain imaging studies in humans and neuroanatomical tract tracing studies in rodents to identity and describe core components of this higher brain circuitry (Figure 1).

### Functional brain imaging experiments of the urge-to-cough in humans

In 2007 our group published the first description of the neural substrates of the urge-to-cough in humans [13] and revealed the complexity of the distributed neural circuitry that detects and responds to airway irritations. In this study we performed functional brain imaging on ten healthy participants during inhalational challenge with a nebulized capsaicin solution, the concentration of which was titrated for each individual to produce a modest urge-to-cough without evoking reflex coughing. The results defined core network components that make up the higher brain sensorimotor control of cough including widespread cortical and subcortical activations that encompass sensory, motor, premotor and limbic structures. In a follow up study [12], we manipulated the intensity of subjects’ urge-to-cough using graded capsaicin challenges, the results of which confirmed the basic core network described in our first study but also enabled us to dissect this distributed network into components (modules) that encode sensory, cognitive or motor responses (Figure 1).

The “*sensory module*” is composed of brain regions that receive (directly or via relay) ascending inputs originating from the airways and encodes either sensory discrimination (stimulus intensity and perception) or spatial discrimination (stimulus localization). Our data suggest that the primary somatosensory cortex and anterior insula play important roles in sensory discrimination whereas the posterior parietal cortex and dorsolateral prefrontal cortex are important for spatial discrimination [12]. These basic patterns of activation are not dissimilar to that described for other sensory modalities of somatic or visceral origin [19,20]. Interestingly, the magnitude of a delivered stimulus may not always be reflected in an individual’s perception of the stimulus magnitude. Indeed, other cortical functions including emotion, attentional focus and alertness can influence the perception of stimulus intensity [17,21,22]. By taking advantage of this, we have noted distinctions between those brain regions (e.g., the anterior insula) that faithfully activate in a stimulus-dependent fashion versus other regions (e.g., primary sensory cortex) where the activation correlates with how intense subjects perceive the stimulus to be [10,12]. We interpret this as evidence for distinct components of the sensory discrimination network, one of which decodes stimulus intensity and the other incorporates this information with other competing central processing to generate the magnitude of the perceivable urge-to-cough. Urge-to-cough experimental paradigms, like other examples of experiments employing noxious stimuli, recruit orbitofrontal cortex, cingulate cortex and other limbic regions (the “*cognitive module*”) which are likely involved in shaping an individual’s affective responses to airway irritation [23,24]. Less is known about the affective processing associated with airways irritation. Of interest, however is that individuals with respiratory disorders such as asthma or chronic cough have a significantly increased risk of developing mood and anxiety disorders, and it seems likely that this would be reflected in altered activity of the network components comprising this cognitive module [25-28].

Many of the network components identified in our urge-to-cough fMRI studies include regions that are activated in other sensorimotor paradigms, including noxious stimulation of cutaneous tissues and other visceral sensory modalities (studies of pain, dyspnea or esophageal distension, for example) [10,29-32]. This suggests the existence of a core network in the brain that plays a generalized role in interoceptive processing. However, given that each of these sensory stimuli enlist distinct sensations and different behavioral responses, the core network must be either topographically arranged or be supplemented by additional neural components that allow for tissue specific responses. Identifying such functional elements within this network is a significant challenge.

### Functional brain imaging experiments of cough motor control in humans

Given that a primary purpose of the urge-to-cough is as a sensory experience to promote behavioral modifications in respiratory control (i.e., to facilitate or suppress coughing), it is therefore of interest to understand the higher brain circuitry responsible for voluntary control of cough. We and others have performed studies of voluntary cough, capsaicin-evoked cough and/ or cough suppression during capsaicin inhalation to define the components of the cough “*motor module*” [7,11]. Voluntary cough is associated with activity in a number of regions including the sensorimotor cortex, supplementary motor area and cerebellum. A distinction can be made between voluntary cough and reflex cough by the pattern of activation in the posterior insula and posterior cingulate cortices that is characteristic of capsaicin-evoked cough, indicating that the brain activity associated with reflex cough is not simply a function of that produced by voluntary cough and airways irritation [7]. The distinction between reflex and voluntary cough is also apparent at the brainstem level. Thus, whereas reflex cough is associated with medullary activation, there appears to be minimal brainstem involvement associated with voluntary coughing [7]. We have interpreted this finding as evidence that corticospinal pathways may be responsible for voluntary coughing, rather than cortical inputs into the medullary respiratory circuit per se [33]. The suppression of irritant evoked coughing is also associated with a unique pattern of brain activity, including an involvement of the anterior insula, supplementary motor area, motor cingulate cortex and right inferior frontal gyrus [7,12]. Interestingly, the right inferior frontal gyrus, along with the pre-supplementary motor area, prefrontal cortex, subthlamamic nucleus and basal ganglia, comprises an inhibitory network that has been shown to be involved in response inhibition during a variety of motor suppression paradigms [34,35]. Comparable activations are also associated with volitional breath holding, supporting the notion that this circuitry is intimately involved in respiratory and cough suppression [36].

### Neuroanatomical organization of ascending airway afferent pathways

The functional brain imaging studies described above provide a ‘snapshot’ of the brain regions activated by a given respiratory task. However, they do not necessarily detail the pathways via which these regions are interconnected. We have begun to assess the anatomy of the ascending circuitry that arises from the airways and provides input to the cortex using a novel viral neural tracing system. To do this, we have developed recombinant herpes simplex viruses (HSV1) strain H129 that genetically encode for the production of green or red fluorescent proteins in infected cells [37,38]. HSV1 H129 is unique in that it is one of only a few viruses that have the ability to infect neurons and pass between synaptically connected neurons in the anterograde direction [37,39]. This property makes the H129 virus ideal for tracing sensory neural pathways and following their connectivity deep into the brain.

**Figure 2:**
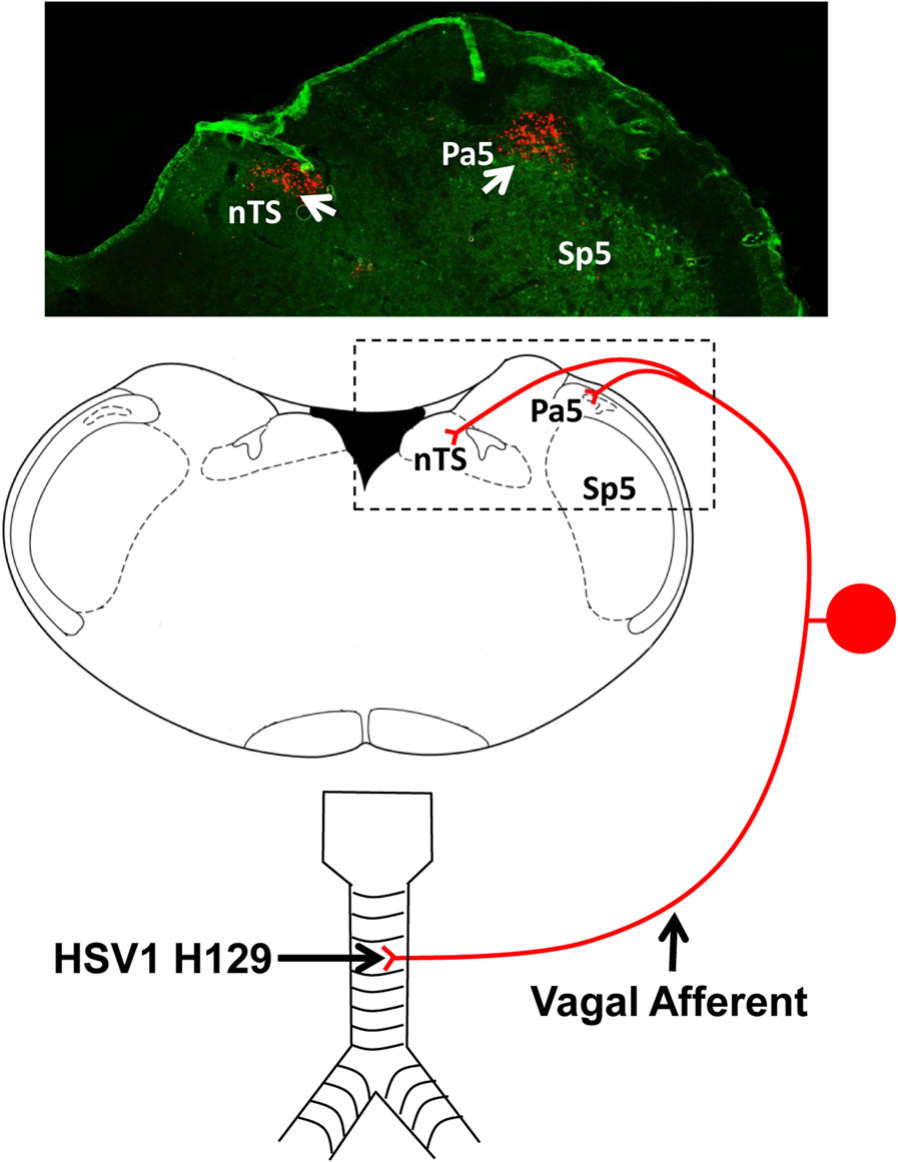
**Mapping the brainstem terminations of tracheal and laryngeal afferent fibers using neurovirulent viruses.** The schematic diagram shows herpes simplex 1 strain H129 tracing of tracheal vagal sensory neurons in rodents involved in sensing airway irritations. Note that afferents terminate in both the nucleus of the solitary tract (nTS) and the paratrigeminal nucleus (Pa5). The photomicrograph above shows an example of nTS and Pa5 terminations traced from the airways using a recombinant HSV1 H129 expressing a red fluorescent protein. See text and references [37,38] for further details.

**Figure 3:**
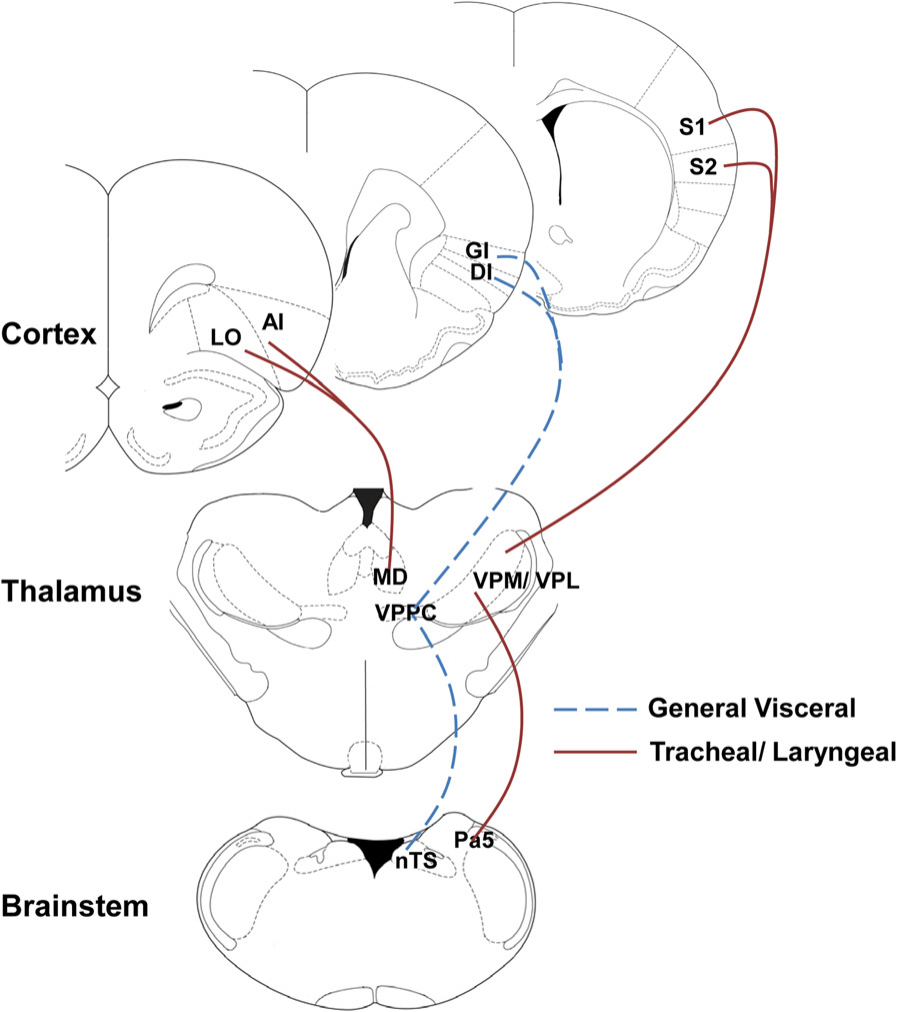
**Putative ascending circuitry for visceral and airway sensations.** Previous studies [41] have shown that general visceral afferents project from the nucleus of the solitary tract (nTS) to the visceral sector of the thalamus (the ventral posterior parvocellular nuclei, VPPC) and onto granular and dysgranular insula cortices (GI and DI, respectively). By contrast, we propose that trigeminal (specifically Pa5) neurons receiving inputs from the trachea and larynx project to the ventral posterior thalamic nuclei (VPM/ VPL) and onto primary and secondary sensory cortices (S1 and S2, respectively). This trigeminothalamocortical pathway may be particularly important for encoding sensations arising from the airways. In addition, projections from the mediodorsal thalamus (MD) to the agranular anterior insula and lateral orbital cortices (AI and LO, respectively) likely encode affective responses associated with airways irritation. Omitted for clarity are the pontine relay nuclei which receive inputs from the medulla and project onto the VPPC and MD. See references [37,38] for further details.

Using recombinant HSV1 H129 in rodents we have shown that tracheal afferent neurons terminate in two brainstem nuclei, the nucleus of the solitary tract and the trigeminal/ paratrigeminal nuclei (Figure 2). Whilst previous studies have documented in some detail tracheal afferent terminations in the nucleus of the solitary tract [9,40], there haven’t been any previous reports of tracheal sensory neurons projecting directly to the trigeminal regions of the brainstem. At present it is unclear of the functional significance of vagal afferent innervation of trigeminal neurons nor is it clear whether it is a specific subset of airway afferent nerves that projects to the trigeminal nucleus or if all airway afferents provide collateral terminals to this brainstem region. However our data would suggest that a significant population of trigeminal neurons may relay airway afferent input to thalamic loci, likely via well described trigeminothalamic tracts [37]. Indeed, one population of thalamic relay neurons is located in the ventral posterior nuclei, but not in the visceral sector (the ventral posteriolateral parvicellular thalamus as defined by [41]), suggesting that upper airway ascending pathways more resemble those of cutaneous afferents rather than other vagal afferents. These neurons in turn project onto layer IV and V primary and secondary somatosensory cortices, which again are in distinct loci when compared to the cortical terminations described for other visceral and vagal systems [41,42] (Figure 3). We speculate that trigeminothalamocortical pathways may prove to be an important circuitry encoding perceptual awareness of airway irritation, and hence in the generation of the urge-to-cough. A second group of thalamic relay neurons is located in the dorsomedial thalamus and in turn send projections to limbic regions of the brain including the anterior insula and orbital cortices [37,38]. Recently Davenport and colleagues [43] reported that tracheal occlusion in rats leads to altered gene expression in the medial thalamus and suggested that this was due to inputs from respiratory mechanoreceptors. These data support our assertion that the medial thalamus is also involved in encoding airway sensory input, although it seems likely that this relates to affective processing relating to anxiety, fear and arousal involving the limbic brain as opposed to sensory perception or discrimination per se [37,38,43]. Importantly, these basic anatomic circuits identified in rodents are in close agreement with our functional brain maps generated from fMRI in humans.

## Concluding remarks

There is still much to learn about the basic organization of the suprapontine brain circuits that detect and respond to airways irritation. Our understanding of the anatomy, physiology and pharmacology of these circuits is in its infancy. Furthermore, whether patients with a chronic cough disorder display functional changes in these central neural processing sites is not known. We propose that aberrant cough associated with airways disease may indeed be associated with abnormal processing in the brain networks involved in sensory perception of airways irritation and/or motor suppression of cough. If correct, this would provide an alternative therapeutic goal for treating chronic cough. Restoration of normal central processing could allow for cough normalization in disease without disrupting either voluntary or reflexive cough, which are essential for the protection of the airways during occasions of acute insult.

## Competing interests

The authors declare that they have no competing interests.

## Authors’ contribution

AM, SY, AW, SP carried out the animal tracing studies while AA and JL carried out the human imaging studies described in the manuscript. SM and MF analysed and interpreted the data. SM drafted the manuscript. All authors read and approved the final manuscript.

## Funding

The data described in this manuscript was funded by grants (1025589, 1042528) to SBM and MF from the National Health and Medical Research Council of Australia.
